# Interaction Empowerment in Mobile Health: Concepts, Challenges, and Perspectives

**DOI:** 10.2196/32696

**Published:** 2022-04-13

**Authors:** Marietta Hamberger, Nensi Ikonomi, Julian D Schwab, Silke D Werle, Axel Fürstberger, Angelika MR Kestler, Martin Holderried, Udo X Kaisers, Florian Steger, Hans A Kestler

**Affiliations:** 1 Institute of Medical Systems Biology Ulm University Ulm Germany; 2 Internal Medicine I University Hospital Ulm Ulm Germany; 3 Department of Medical Development and Quality Management University Hospital Tübingen Tübingen Germany; 4 Chief Executive Officer University Hospital Ulm Ulm Germany; 5 Institute of the History, Philosophy and Ethics of Medicine Ulm University Ulm Germany

**Keywords:** mHealth, mobile apps, patient empowerment, digital health, interaction empowerment, patient-doctor relationship, health care network, intersectoral communication

## Abstract

In its most trending interpretation, empowerment in health care is implemented as a patient-centered approach. In the same sense, many mobile health (mHealth) apps are being developed with a primary focus on the individual user. The integration of mHealth apps into the health care system has the potential to counteract existing challenges, including incomplete or nonstandardized medical data and lack of communication, especially in the intersectional context (eg, patients, medical forces). However, concerns about data security and privacy, regional differences in regulations, lack of accessibility, and nontransparent apps hinder the successful integration of mHealth into the health care system. One approach to address this is to rethink the interpretation of empowerment. On that basis, we here examine existing approaches of individual empowerment and subsequently analyze a different view of empowerment in digital health, namely interaction empowerment. Such a change of perspective could positively influence intersectoral communication and facilitate secure data and knowledge sharing. We discuss this novel viewpoint on empowerment, focusing on more efficient integration and development of mHealth approaches. A renewed interpretation of empowerment could thus buffer current limitations of individual empowerment while also advancing digitization of the health system.

## Background

Mobile health (mHealth) represents reformed health and medical care achieved through mobile devices. In the clinical setting or outside hospital walls [1], mHealth is meant to improve and support the prevention, diagnostics, therapy, monitoring, and follow-up care of and with patients [2-5]. Infomediaries, mobile apps, telemedicine, and mobilized medical devices are examples of how mHealth has already been introduced into medical care [3,6-10]. Despite these benefits, limitations due to security concerns, possible health risks, corresponding regulations, and individual barriers influence the development of mHealth and its integration into existing clinical structures [11-20].

**Figure 1:**
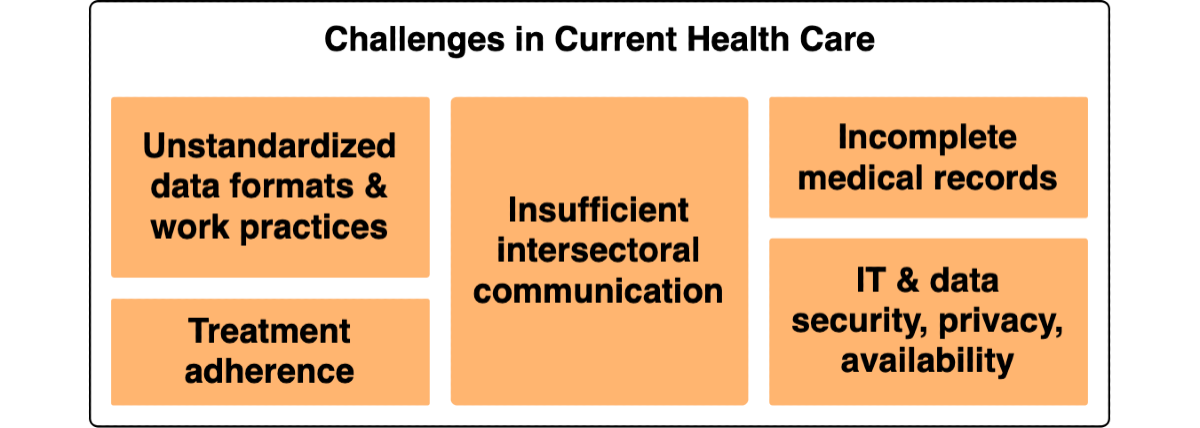
Major challenges in the current health care system. IT: information technology.

Within current clinical settings, common challenges are incomplete medical records [21], data security and privacy [22], as well as unstandardized data formats or work practices [16,23]. Furthermore, missing treatment adherence [24-26] and insufficient communication among physicians, patients, and other health care providers (HCPs) may impede medical care [27-29], as shown in Figure 1.

To address these challenges, we here provide a new viewpoint on the inclusion of empowerment concepts when developing mHealth apps. By summarizing current concepts and challenges in digital medicine, we provide solutions and examples on how to ensure an interaction network–based view on empowerment. Our viewpoint attempts to encourage new discussion and perspectives in implementation of mHealth apps within the current complex health care network of involved parties.

## Context and Challenges in mHealth Apps

mHealth inter alia aims at improving the challenges of current health care. Through mHealth, various health conditions such as chronic diseases [30], acute conditions [1], mental health disorders [10], or nonillness-related motives are addressed [6]. Many of the current approaches mainly focus on empowerment of the individual person, especially on patient empowerment. For instance, appointment managers [9] and infomediaries already exist for enhanced (educational) communication [8]. Moreover, patient-managed symptom and medication diaries [7,10] or implemented notifications regarding important events are used to improve treatment adherence or counter incomplete medical records [10]. Although the responsibility for treatment decisions for children remains with parents or guardians, this integration process aims to accomplish informed consent by involving minors in the decision-making process [7,30].

**Figure 2:**
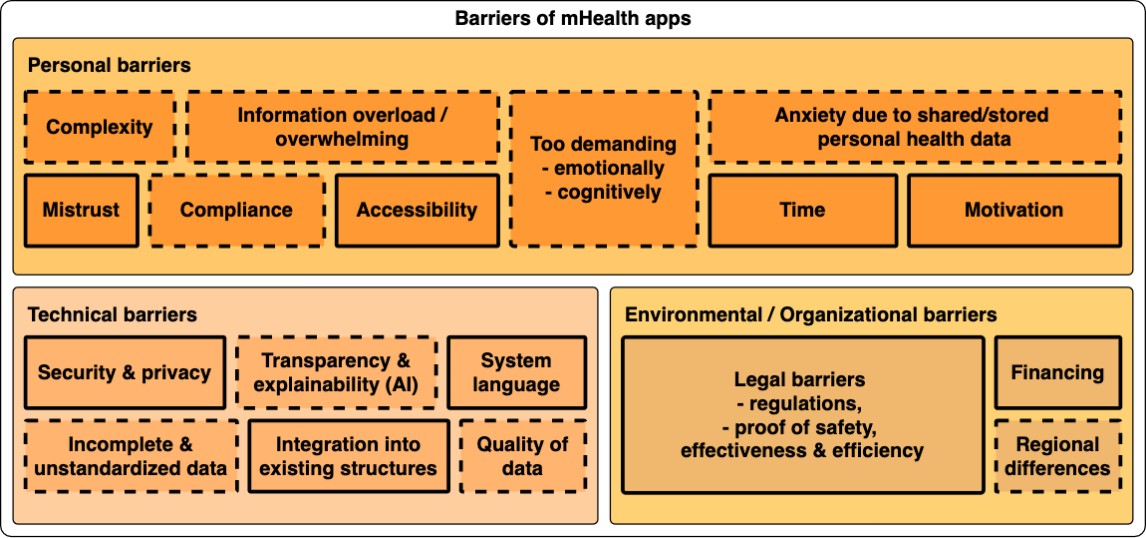
Three main classes of barriers in designing mobile health apps as categorized by Schreiweis et al [31]. For each larger class, subboxes including specific issues are depicted. Boxes with solid borders represent barriers mentioned by Schreiweis et al [31]. Additional or more detailed hurdles we collected from the literature are highlighted by dashed lines [11-20]. The size of the boxes does not represent their relevance or severity. AI: artificial intelligence.

Nonetheless, mHealth approaches also entail further barriers that can roughly be separated into personal, technical, and environmental and organizational barriers, as concluded by Schreiweis et al [31]. In Figure 2, we extend this categorization by incorporating additional hurdles collected from the literature [11-20]. While some barriers might hinder the individual user from being empowered, technical or environmental and organizational barriers might influence the necessary interactions among those individuals. As an example, missing standards might obstruct interaction among physicians, developers, and researchers. On the other side, mHealth could also simplify the introduction of standardized practices in health care. However, the actual use would depend on the willingness of individual organizations or people.

Moreover, the sheer number of existing standards and their regional differences may further compromise this approach [32]. The internal competition between the quality of the service and the financial expenses might become disadvantageous for the patient, which is especially relevant in countries dominated by privatized health care services (eg, the United States) [33,34]. Given the rising costs of digitalization processes in health care services, mHealth apps can become a key point on future reduction of financial efforts. In this direction, open-source apps can help in reducing the overall expenses for individual institutions [35].

Although numerous mHealth apps are now freely available for download in leading app stores, only a handful have been designed in accordance with current regulations or guidelines or have been approved by an ethics committee [36,37]. Aside from significant security concerns, this may lead to discouragement among users, who may not be able to differentiate which services best suit their needs [36].

Another obstacle to the global health system benefiting from digitalization is the scarcity of evidence reporting on mHealth interventions [38,39]. Adding to this difficulty, the few existing reports display a broad variety of quality, detail, and objectivity.

To enable accurate evaluation and reproducibility, standardized reporting should also be considered when developing and testing mHealth strategies. The World Health Organization (WHO) mHealth Technical Evidence Review Group devised a checklist for reporting and assessing mHealth evidence. In this checklist, the intervention’s scope and context, as well as its technical foundation, may be outlined in a predefined structure [37].

In addition to costs and infrastructure organization issues, most of the challenges in integrating health care and mHealth approaches rely on the involvement of stakeholders. A classical view of the mHealth development and implementation process sees patients as the primary users and, therefore, the empowerment focus. However, in a more realistic scenario, all of the components in the surrounding health care system should also be considered [40-42].

**Figure 3:**
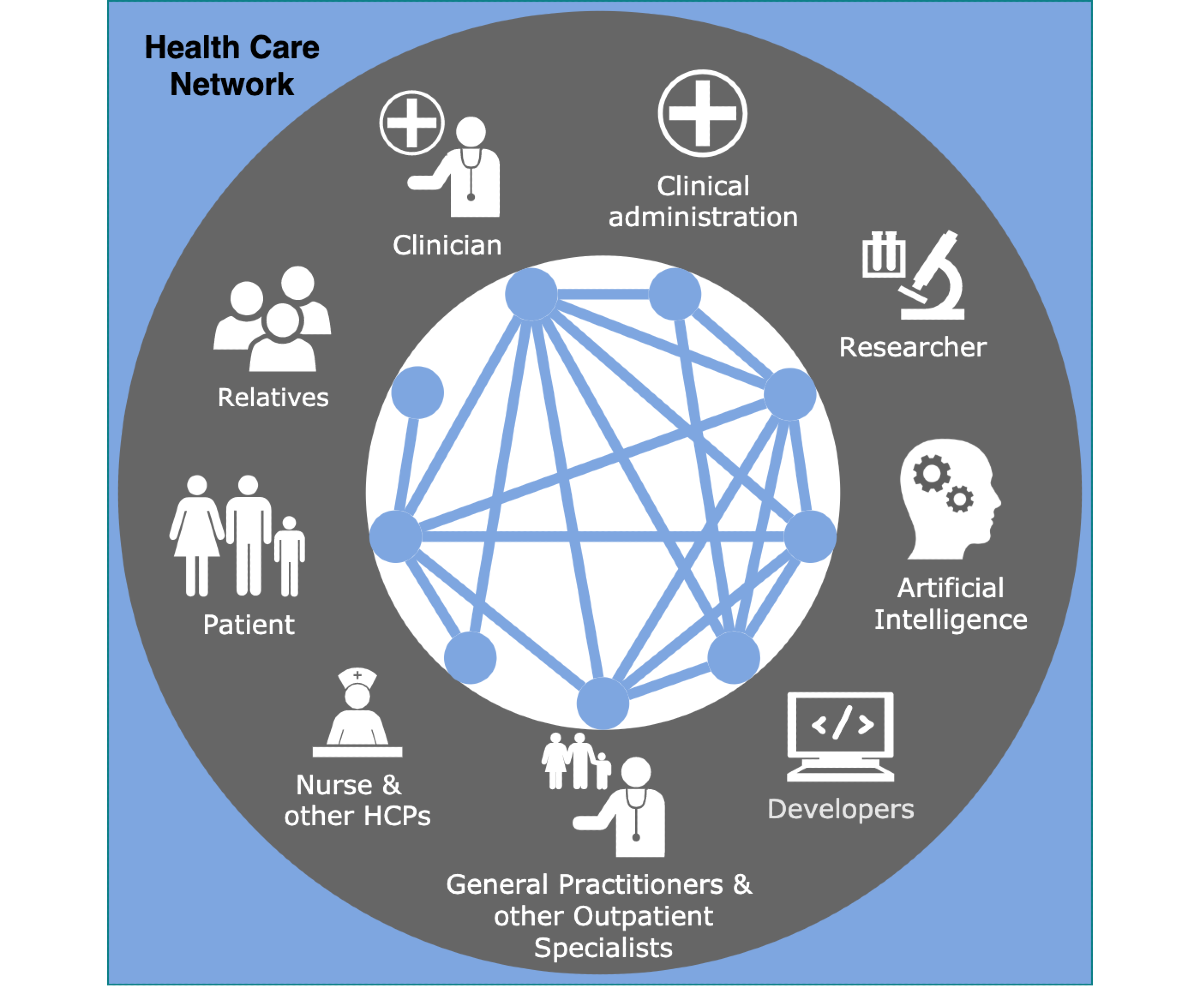
Representation of the modern health care network. Each node represents a relevant group of individuals interacting within this network with other individuals. Connecting edges represent these possible interactions. Even though there are more actors in the existing health care system, we merely included the groups we evaluated to be the most relevant for the concept of empowerment. HCP: health care provider.

The network underlying the current health care system comprises different roles. As depicted in Figure 3, a representation of this network can be a complex graph with many participants and interactions. Many representations of the health care system depict the patient in the center or do not allow for an iterative information flow due to a one-directional or tree-like architecture [43,44]. Despite the fact that the patient’s well-being is of paramount significance, we wish to emphasize the importance of *all* actors and their interactions via this decentralized interpretation. Toward this end, we adopted a circular design in an attempt to display the relevance of each participant in a more balanced manner.

In the following, the term “node” is used to refer to a specific group of individuals, such as physicians, while “edges” describe the node’s interaction (eg, the patient-physician relationship). The edges in Figure 3 represent the direct interactions that we evaluated as relevant, meaning that we excluded, for example, the interaction between relatives and developers due to the uncommonness. Nevertheless, the interaction between patient and relatives may play a role in health care since relatives may be able to observe significant events or provide emotional support. Here, and in the following, we consider two types of relatives. On the one hand, we refer to close contacts, as they are involved in the supportive care of patients among other roles. On the other hand, we consider legal guardians of a patient if they have the final decision-making power, as in the case of children. To simplify this separation, we use the term “relatives” to refer to the first group and the term “patient” to denote the individuals making the decision (ie, a legal guardian), if applicable.

## Empowerment of Individual Nodes in the Health Care Network

### Overview

Ideally, the patient’s health should be seen as the main objective of the health care network. Nonetheless, each actor may follow their own personal objective while trying to gain power and fearing losing it while providing or receiving health care.

### The Patient

We assume that the patients’ primary goals are to maintain or improve their health, manage their lives, and receive adequate support after receiving a diagnosis. A hierarchical or linear organization of health care (ie, putting the more medically educated HCPs at the beginning or top and the patient at the receiving end) might be outdated. Recently, patient-centered care has been considered to be more beneficial [45]. Given the trend of patient empowerment, it is natural to picture the empowered patient in the center with supporting individuals and HCPs acting in the surroundings. Regarding shared decision-making, most patients prefer to be included in the decision-making process.

Nonetheless, approximately half of all patients would prefer that the physician should have the final say, even though this case is legally unenforceable since physicians are merely acting on behalf of the patient. Therefore, the average patient strives to be included in the decision-making process, yet the preferences regarding the final decision would theoretically vary [46]. Furthermore, the patients’ preference for playing an active role in the treatment process depends on different factors, including their age [46], ethnicity, or level of education [47]. Some patients do not want to be confronted by their health condition or do not feel that they are sufficiently ill to take action, leading to a potential delay in reaching out to HCPs [48-50]. Hence, patients may differ in their preferences concerning decision-making or being educated. Nevertheless, the patient, or their legal guardian, is obligated to decide, which emphasizes the importance of sufficient education and information.

### Artificial Intelligence

In Figure 3, we also included artificial intelligence (AI) as a relevant interacting node. Recently gaining popularity, AI is already part of various health-related apps and devices [51]. AI may also be integrated into mHealth, yet current algorithms are still too prone to errors. Hence, at this point in development, they cannot be trusted with medical decisions (eg, due to not being able to interpret social cues or engaging in personal discussions with the respective patient) [52]. Nonetheless, the continuous improvement of such apps justifies the use of AI in an assisting manner. In this context, the transparency and explainability of these systems is crucial to ensure human users’ trust in AI as assistants [52,53]. An explainable app assists the users and reveals why it made a specific assessment. A user with domain knowledge can then interfere and perform error correction in case of erroneous results. This approach may also help to detect uncommon lesions, which might be overseen due to the inexperience of the individual clinician.

Moreover, the AI screening approach may save time for the clinician, which can be redirected to patient consultation and more direct contact. Using a conversational interface, chatbots can, for example, provide an initial evaluation of a patient’s mental or physical state or empower individuals confronted with a life-altering condition [54,55]. Besides alleviating the frequent concern of patients to lose personal contact due to digital medical approaches [56], such approaches linking the AI to the clinician can further boost the doctor-patient relationship. Moreover, while the user benefits from an AI assistant without giving up control, the AI benefits from the chance of improvement through error correction [53]. In this sense, this interaction can be seen as mutually empowering if accessibility is put into practice to counterbalance social inequalities. Yet, it is essential to note that additional challenges accompany the benefits of AI integration. For this purpose, the WHO guidance on Ethics & Governance of Artificial Intelligence for Health promotes an ethical integration of AI into digital health care solutions based on six core principles [57], including discussing the transparency of AI in health care. Additionally, biased databases are problematic since they are often based primarily on male subjects and usually do not include minorities, possibly limiting the validity of the data [58,59].

### Health Care Providers

Physicians, either practicing in primary care or a clinical environment, must still focus on compassionate healing as the foundation of health care, even though the mode of interacting may change. Frequently, continuity of care is impeded by insufficient data sharing among physicians and other HCPs (eg, information about treatment decisions). Simultaneously, being equipped with more time, opportunity, information, and technologies, the training of the empowered physician to treat and monitor remotely is also essential [17]. Furthermore, the voice of nursing and the viewpoint of frontline workers is described as a critical point in developing mHealth apps [18]. In this context, the empowerment of nurses and community health workers (CHWs) can positively transfer to their patients [9,18].

## Problems of Empowering the Individual in mHealth

Even though the traditional doctor-patient relationship may leave the patient with less control and provided knowledge, patient-centered health care combined with the hard-to-balance empowerment of individual roles can result in a fight-or-flight reaction among physicians. While in this movement, the patient-physician relationship changes into a partnership, with the physician’s role slowly shifting into guiding the patient in shared decision-making [17], physicians might see their clinical autonomy threatened. However, the patient’s trust in digital solutions may be influenced by the physician’s opinion and recommendation [11,60]. In addition, personal factors can influence mHealth app usage, such as age, general preferences, or educational background [61]. The resulting physicians’ disempowerment can thus counter the anticipated advancements. Thus, the empowerment of an individual node may positively influence the empowerment of another in the health care network. Nonetheless, the exact opposite now poses a parallel challenge, where empowerment of one individual triggers disempowerment of the other. Additionally, the individuals’ empowerment through mHealth does not directly tackle the challenges of current health care mentioned above (see Figure 1).

Even though individual empowerment might positively influence treatment adherence, the other challenges remain primarily unaffected. The reason for this may rely on the fact that these other problems emerge when individuals are interacting (eg, while communicating, through data exchange, or teamwork). Since the approach of empowering the individual merely focuses on the network’s nodes, the edges in between nodes are not prioritized. Thus, instead of empowering single nodes of the health care network, we recommend strengthening the interactions, thereby empowering the edges. This change of viewpoint may enhance the ability to develop approaches tackling the interaction-related challenges.

## Empowerment of the Interaction Network

Based on the issues mentioned above, we propose a new view on empowerment, namely the *empowerment of interactions*. All interactions follow a specific goal that is mostly shared among all participants, such as the improvement of a patient’s acute health status or the development of an app [62].

Through a pooled skill set and empowered mutual support, the underlying goal might be reached more efficiently. Instead of trying to improve each individual’s skill set, the weaknesses of one party could be directly balanced out by the strengths and skills of another [63]. Hence, technical skills, medical knowledge, information about patient history, administrative and legal regulations, and standard practices can be actively shared at the point of need, in contrast to time-consuming preeducation. Here, in empowering the interaction, the redistribution of power and control, as well as the reshaping of single relationships, is not essential yet possible. As an example, privacy and security issues have to be considered within their network of influence. For instance, by empowering communication between developers and HCPs in defining safety priorities, patients will also benefit in trust when using the mobile app. Hence, instead of a technical implementation, data security becomes a key factor within empowering trust and safety of involved parties [64].

Thus, this approach can facilitate integration into traditional structures and offers the benefits of individual empowerment, such as better-informed patients or improved continuity of care.

Until this point, we have considered a general network of multiple individuals with specific roles that interact with others in various ways, all depending on the interaction’s goal. Nevertheless, the concept of interaction empowerment is applicable on multiple levels. For example, the simplest way would be to empower the relationship between two individuals. Yet, on a more abstract and broad level, one could empower multiple interactions simultaneously. In pooling single interactions together to form subnetworks following a common goal, we can again focus on one entity for empowerment, namely the subnetwork.

## Interactions in the Health Care Network

### Defining Subnetworks

Various interactions take place in the health care network (see Figure 3). Within the network, individuals communicate, share data and knowledge, cooperate, or provide treatment and support [65,66]. While interacting, each individual has a specific role comprising their own skill sets, motives, and more personal characteristics. Additionally, the same individuals may interact in different ways based on their goal of interaction. Hence, the individuals’ roles and interactions may change depending on the underlying interaction type and intention (eg, primary care or research and development) [67,68]. Exemplarily, a physician might be treating a patient (primary care), whereas in another context they can contribute to research projects (development). While the primary aim relies on empowering the network of stakeholders, single nodes still retain decision power on downregulating or eliminating a specific interaction. This implies that individual nodes (stakeholders) and edges (empowerment means) can crosstalk in defining the final empowerment network.

Empowering the interaction does not merely mean reshaping or prioritizing relationships. Instead, such empowerment can be defined as identifying the therein present interactions, their underlying goals, and relevant actors, and determining how these interactions can be supported in a balanced manner. Given the multilayers and roles described above, the overall interaction network could end up being a complex interplay of intricate edges. Hence, we propose to approach interaction networks merely as a channel for information flow, independent of the types and number of participants. This allows for the formation of generalized constructs, which can be resolved in generating functional subnetworks for each empowerment task. For this purpose, we next aim at partitioning the health care network into functional subnetworks.

**Figure 4:**
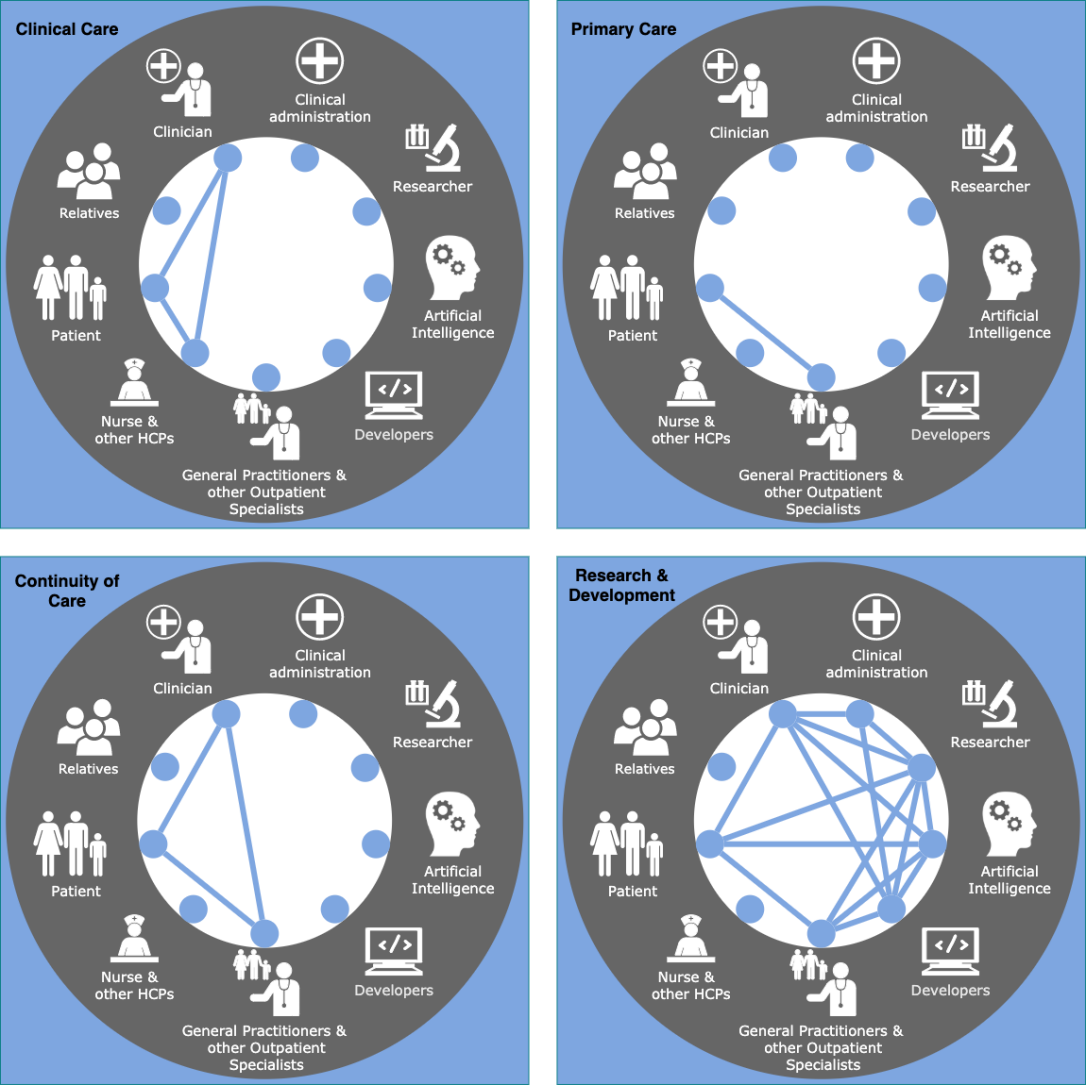
Four examples of possible subnetworks in the modern health care network. Each node represents a relevant role (ie, individual or group) and the connecting edges represent relevant interactions in the underlying subnetwork. The top left caption describes the interaction’s main aim. HCP: health care provider.

Putting these concepts into practice, in principle, almost all nodes of the health care network may interact with each other for multiple contexts and goals (see Figure 3). Following the idea of the communication flow, subnetworks can be identified based on specific tasks (see Figure 4). Subnetworks can overlap in part of their nodes and interactions, still yielding a different empowering task. In addition, one subnetwork may be entirely part of another subnetwork regarding their nodes and edges, yet aiming at another or more specific goal. Exemplarily, the primary care subnetwork and the continuity of care subnetwork share common nodes and edges, even if the final empowerment task is set to different aims.

Through pooling instances (ie, nodes and edges following the same goal), their weaknesses may be balanced out by the strengths and skills of other participating individuals. With this approach, the mentioned benefits of interaction empowerment are still incorporated, yet the count of empowerment targets is reduced to the count of chosen subnetworks. In summary, we treat the subnetwork as one instance following one goal with a set of strengths and weaknesses that may cancel each other out to a certain degree.

### Use Cases of Subnetworks in Health Care

To provide practical use cases, we describe four chosen subnetworks, as depicted in Figure 4: clinical care, primary care, continuity of care, and research and development. To each subnetwork, we map the challenges of current health care that we assume to be positively influenced by it.

In the first subnetwork, patients, clinicians, nurses, and other HCPs interact for clinical health care. Here, lacking treatment adherence might obstruct successful clinical care. The empowerment of this network might help to tackle this challenge.

Similarly, the patient may interact with the family physician for primary care (ie, for day-to-day health care). Here, the lack of treatment adherence also poses a challenge. In this specific case, the network generally only comprises two nodes with one interaction entity. We nonetheless consider this interaction as relevant to form an individual subnetwork.

Third, the interaction among clinicians, the family physician, and the patient ensures continuity of care, as in the context of a hospital stay. However, unstandardized data formats and work practices, data security, insufficient intersectoral communication, and resulting incomplete medical records hinder the continuity of care. These challenges may, in our opinion, be tackled by empowering this subnetwork.

Finally, in research and development, researchers, developers, physicians (clinicians/family physicians), patients, and clinical administration may interact, with each node’s contribution and degree of involvement depending on the unique context/setting. Here, the researcher, developer, or clinician might present as the same individual, such as in the field of personalized medicine. Academia-industry cooperation and institutional review boards can also fall into this fourth category. Herein, the progress is slowed down by data security and privacy issues and insufficient intersectoral communication. Again, by empowering this subnetwork’s interactions, we argue that those challenges may be improved.

### Empowering Subnetworks Through mHealth

Some mHealth apps already support a concept of empower-oriented communication through interactions. In the following, we summarize some examples tackling different network empowerment concepts, which can be used as a starting point for further development.

CoCoV [69] is an mHealth app that supports patients to track their symptoms after receiving a COVID-19 vaccination through an easily understandable questionnaire. This app empowers the physician-patient interaction in preventing lost or overlooked symptoms. Additionally, the safely and anonymously transferred structured data can further be collected, enabling enhanced analysis of adverse symptoms by researchers and data scientists. Hence, the design of CoCoV simultaneously tackles two different empowerment subnetworks.

The app NEMO [69,70] supports patients receiving tumor therapy to protocol-related side effects in a structural and standardized manner. Typically, patients may forget to record incidents or may not consider them relevant [71]. The management app hence empowers the patient-physician interaction and communication in counteracting those issues. Since patients can administer the data sharing through QR codes, they can be empowered individually and continuity of care can be enhanced. NEMO [69,70] can be further used to empower the treating physician and other HPCs by facilitating data sharing (eg, among family physicians and specialists). Additionally, as with CoCoV, standardized and anonymized data collection empowers research and development.

Track Your Tinnitus is another symptom tracker enabling the patient to record daily tinnitus perception [72]; the app PainBuddy focuses on pain and symptom management of children with cancer [7]; and MindFrame empowers patients recently diagnosed with schizophrenia in managing symptoms, medication, and recurring behaviors [10]. Like CoCoV and NEMO, these apps empower the individual yet also support the interaction, communication, and data transfer, resulting in interaction empowerment of the respective subnetworks. Focusing more on empowered communication, Ramukumba et al [9] presented an mHealth app used by CHWs. Here, the support for communication through information exchange and timely decision support at the point of care resulted in the empowerment of CHWs, and consequently that of their patients.

FeelBack is a project assessing psycho-oncological stress through an app-based questionnaire. Here, not only patients can be targeted but also their relatives feeling burdened by the illness. Hence, by empowering the related individuals, the supportive interaction among patients and others can be empowered [19].

In simplifying intersectoral communication and data sharing, the browser-based system CTest enables tracking clinical samples online [73]. The tested individual can access their test sample’s status update through personalized QR codes. With this approach, clinical staff can be disburdened while the testee is not dependent on clinical staff for accessing test results. Hence, this interaction is empowered through simplification of the overall communication process.

## Conclusion

Recently, mHealth apps are being developed to aid with specific tasks in the health care system. The empowerment of the individual (ie, patient or physician) is often seen as a central aspect. Nonetheless, this approach entails further challenges and barriers such as disempowerment through empowerment, difficulties in integrating mHealth into existing structures, or social inequalities. We have presented an updated approach to instead focus on the *empowerment of interaction and communication*, since we argue that this could help to tackle current challenges in the health care system even faster and more efficiently. To reach a specific goal (eg, continuity of care), multiple groups in the health care network interact, which can be pooled to a single subnetwork. In developing mHealth with a strengthened focus on empowerment of interaction networks, the individual may still benefit even if the focus is shifted to another level, while also diminishing the barriers of the original individualized approach. Hence, we propose a new perspective on mHealth and empowerment to tackle current health care challenges more efficiently.

## Abbreviations

AI: artificial intelligence
CHW: community health worker
HCP: health care provider
mHealth: mobile health
WHO: World Health Organization
